# Carbon Nanomaterials as Versatile Platforms for Biosensing Applications

**DOI:** 10.3390/mi11090814

**Published:** 2020-08-28

**Authors:** Hye Suk Hwang, Jae Won Jeong, Yoong Ahm Kim, Mincheol Chang

**Affiliations:** 1 Alan G. MacDiarmid Energy Research Institute, Chonnam National University, Gwangju 61186, Korea; 2 Department of Polymer Engineering, Graduate School, Chonnam National University, Gwangju 61186, Korea; sjndow1221@naver.com; 3 School of Polymer Science and Engineering, Chonnam National University, Gwangju 61186, Korea

**Keywords:** biosensors, carbon nanomaterials, functionalization, optical sensors, electrochemical sensors, hybrids, composites, transducers, immobilization

## Abstract

A biosensor is defined as a measuring system that includes a biological receptor unit with distinctive specificities toward target analytes. Such analytes include a wide range of biological origins such as DNAs of bacteria or viruses, or proteins generated from an immune system of infected or contaminated living organisms. They further include simple molecules such as glucose, ions, and vitamins. One of the major challenges in biosensor development is achieving efficient signal capture of biological recognition-transduction events. Carbon nanomaterials (CNs) are promising candidates to improve the sensitivity of biosensors while attaining low detection limits owing to their capability of immobilizing large quantities of bioreceptor units at a reduced volume, and they can also act as a transduction element. In addition, CNs can be adapted to functionalization and conjugation with organic compounds or metallic nanoparticles; the creation of surface functional groups offers new properties (e.g., physical, chemical, mechanical, electrical, and optical properties) to the nanomaterials. Because of these intriguing features, CNs have been extensively employed in biosensor applications. In particular, carbon nanotubes (CNTs), nanodiamonds, graphene, and fullerenes serve as scaffolds for the immobilization of biomolecules at their surface and are also used as transducers for the conversion of signals associated with the recognition of biological analytes. Herein, we provide a comprehensive review on the synthesis of CNs and their potential application to biosensors. In addition, we discuss the efforts to improve the mechanical and electrical properties of biosensors by combining different CNs.

## 1. Introduction

**Figure 1:**
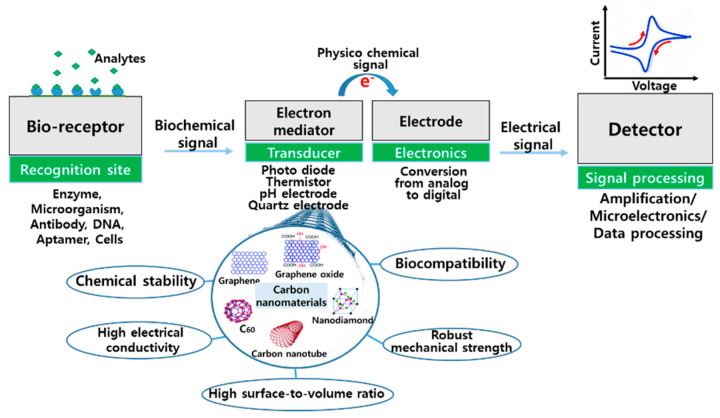
Components and the involved mechanism of a conventional biosensor where carbon nanomaterials (e.g., CNT, graphene, nanodiamond, fullerene) can be used as a transducer.

In recent years, biosensors have become important to modern life because they enable the diagnosis of diseases and the detection of targeted biological agents in the environment [1]. Biosensors are analytical devices that are utilized for the detection of a chemical analyte by converting a biochemical/biological reaction into a measurable physicochemical signal [2,3]. Typically, these devices are composed of a bioreceptor (e.g., enzymes, microorganisms, antibodies, DNAs, aptamers, or cells), transducer component (e.g., semiconducting material or nanomaterial), and an electronic system with a signal amplifier, processor, and display [2]. The bioreceptor specifically recognizes the target analyte, and the transducer converts this response into a different kind of energy that is amplified, processed, and converted into the desired signal format (Figure 1).

From simple biological recognition to complex signal transducing of the interaction between receptors and analytes in sensor platforms, carbon nanomaterials (CNs) have been utilized over the last few decades with a frequent emergence of innovative designs in biosensor platforms. CNs are one of the materials suitable for biosensors because of their attractive features including chemical stability, high electrical conductivity, robust mechanical strength, high surface-to-volume ratio, and biocompatibility (Figure 1). Recently, the use of CNs in biosensor platforms has rapidly grown in the area of biosensor design [4]. In particular, CNs have been used as (i) a recognition element of the sensor, where they provide binding sites for target biomarkers or molecules capturing target biomarkers, (ii) a transducer component that converts the detected molecular interaction on the electrode surface into a measurable signal, and (iii) a label for target biomarkers in signal amplification (Figure 1). The structures of the CNs that are most widely used in biosensors are carbon nanotubes (CNTs), graphenes (GRs), nanodiamonds (NDs), and fullerenes (FRs) (circular diagram in Figure 1) [2]. In addition, some CNs have been shaped into a bare electrode such as a screen-printed carbon electrode that serves the roles of recognition of target analytes and signal transduction in biosensors [5,6].

Functionalization of CNs can open the avenue to a wide spectrum of biosensor applications, due to their unique combinations of chemical and physical properties (i.e., thermal and electrical conductivity, high mechanical strength, and optical properties). In addition, functionalized CNs provide excellent reproducibility with a remarkable specificity and sensitivity to detect target analytes even in very low concentrations. The present review covers all aspects of recent syntheses, composites, and functionalization of CNs for a broad range of biosensor applications.

## 2. Carbon Nanotubes (CNTs)

CNTs present diverse advantages with their unique properties, such as a high surface-to-volume ratio, high electrical conductivity, chemical stability, biocompatibility, and robust mechanical strength [7]. These numerous advantages compared to other currently employed materials offer great promise for CNTs in a wide range of applications, including detection of DNA/RNA, enzymes, and whole cells [4,8,9]. Depending on their number of walls, two basic forms of carbon nanotubes are designated: single-wall carbon nanotubes (SWCNTs) and multiwall carbon nanotubes (MWCNTs). A SWCNT is the rolled form of single graphene sheet, a hollow cylindrical carbon structure that consists of a stable arrangement of carbon atoms linked via sp^2^ bond hybridization. MWCNTs consist of multiple layers of graphite held by van der Waals forces with an interlayer spacing of 3.4 Å [10]. The diameter, curvature, and electronic properties of CNTs are dependent on the arrangement of a hexagonal ring of sp^2^ carbon atoms. The type of carbon hexagonal ring arrangement is referred to as the chirality of CNTs. Based on the orientation of the tube axis with respect to the hexagonal lattice, the structure of a nanotube can be simply defined based upon the chiral vector that is specified by its chiral indices (n, m). SWCNTs are classified by the geometric arrangement of the carbon atoms at the seam of the cylinders; most SWCNTs are chiral (m ≠ n), and some of them present armchair (m = n) or zigzag (m = 0) configurations [11]. MWCNTs can be visualized as concentric and closed graphite tubules with multiple graphene sheet layers; the distance between two layers is very close to the distance between two graphene layers in graphite (~3.5 Å) [12]. Interestingly, CNTs possess an outstanding large surface/volume ratio because of their unique structural configuration.

The high electronic conductivity resulting from the unique structures of CNTs [12] makes them well suited for their use in transducers for biosensors, which convert the recognition of a target into an electrical signal [11]. Moreover, a high specific area with large surface/volume ratio is favorable for immobilizing a greater concentration of functional units on the CNT surface, which detect the target analytes in biosensing applications.

### 2.1. CNT Synthesis

Today, commercially available techniques for fabricating CNT structures [13] are carbon arc discharge [14], laser ablation [15], and chemical vapor deposition (CVD) [16] (Table 1). The high-temperature-based techniques (e.g., laser ablation and arc discharge) were initially used for CNT fabrication. However, currently, these techniques have been substituted by low temperature-based CVD methods (<800 °C) due to their more accurate control of the CNT features such as length, diameter, alignment, purity, and density [17].

**Figure 2:**
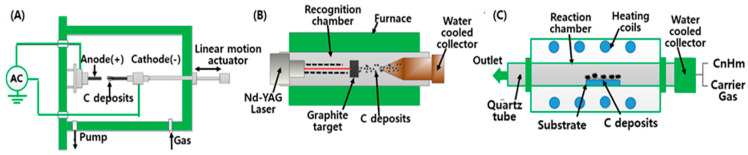
Schematic representation of the methods used for carbon nanotube synthesis: (**A**) arc discharge, (**B**) laser ablation, and (**C**) chemical vapor deposition.

The arc-discharge techniques utilized at higher temperatures (above 1700 °C) exhibit the following merits in the production of CNTs: high yield, better control over the size, and fewer structural defects in comparison with other methods (Figure 2A). High-purity graphite (6–10 mm optical density) electrodes or water-cooled electrodes with diameters of 6–12 mm are used for the arc-discharge methods, separated by 1 to 2 mm in a chamber filled with helium (500 torr) at subatmospheric pressure [18]. Direct current is passed through the chamber that contains a graphite cathode-anode, evaporated carbon, and some amount of metal catalyst particles, while the chamber is pressurized and heated to approximately 4000 K [17]. There are two different methods for the arc-discharge deposition depending on the usage of catalyst precursors. Generally, synthesis of SWCNTs utilizes various catalyst precursors and a complicated anode that consists of graphite and a metal, whereas MWCNTs could be synthesized without the use of catalyst precursors.

In the high-power laser ablation method, a high-power laser (YAG type) is used to vaporize a target of pure graphite with a metal catalyst heated at ~1200 °C in an argon or helium atmosphere (Figure 2B) [16]. Like the arc-discharge method, the addition of metal particles with high purity and high quality as catalysts to the graphite generation is necessary in laser ablation. The principal mechanisms for the synthesis of CNTs in both laser ablation and the arc-discharge technique are similar, while the main energy sources are different in that the former uses a laser and the latter employs direct current. Typically, the CNTs obtained by laser ablation are not necessarily uniformly straight, nor is this method economically advantageous, because it requires a great amount of laser power and high-purity graphite rods, and results in low yields of CNTs.

As one of standard techniques for the synthesis of CNTs, CVD is an economically practical method for large-scale, high-quality CNT production and allows for easy control of the reaction course, which is performed with simple equipment at mild temperature and pressure conditions [17]. The main process of the CVD method is to excite carbon atoms in the presence of metallic catalyst particles (e.g., Ni, Co, and Fe). The initiators of CNTs are deposited on the top surface of the substrate at its bottom, then a hydrocarbon such as acetylene is heated. To initiate the growth of CNTs, a process gas (e.g., ammonia, nitrogen, or hydrogen) and a carbon-containing gas (e.g., acetylene, ethylene, ethanol, or methane) are blended into the reactor. CNTs can grow to be very long and very well aligned on a layer of metal catalyst particles that is prepared on a substrate at approximately 700 °C.

### 2.2. Electrochemical and Electronic CNT Biosensors

By virtue of their electrical and electrochemical properties, CNTs are suitable for integration into electrochemical biosensors for enhancing electron transfer [1,3,19,20,21,22]. As mentioned earlier, CNTs have a small size and large surface/volume ratio with unique physicochemical properties, enabling the immobilization of a large number of functional units such as receptor moieties for biosensing [3,23]. A wide variety of electrochemical CNT biosensors have been developed to detect ions, metabolites, and protein biomarkers [1]. The enzymatic catalysis of a reaction that produces electroactive species can boost the electric signal arising from the electroactive surface area of CNTs. Hence, the target analyte is recognized by the enzymes immobilized on the working electrode, thereby producing a current or contributing to voltage production [11].

**Figure 3:**
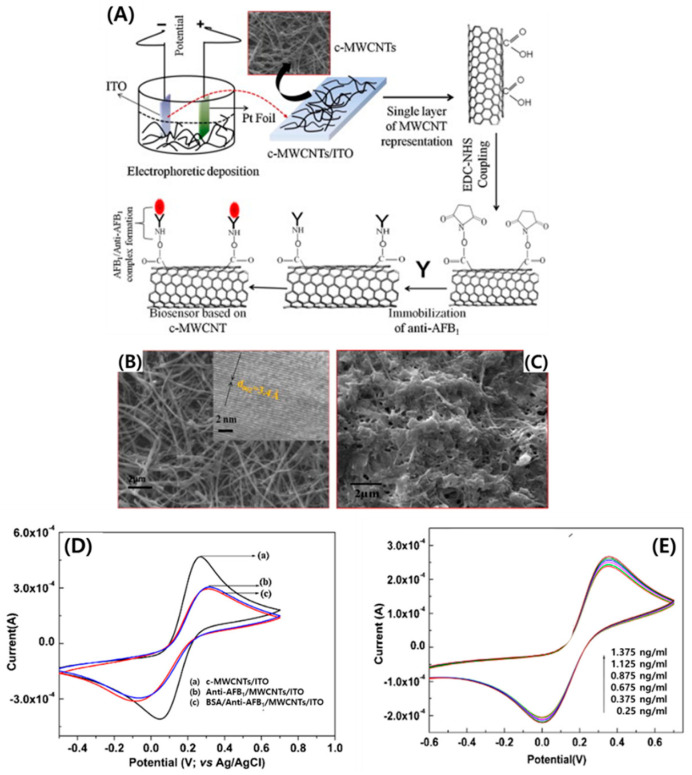
(**A**) Schematic illustration of the electrochemical biosensor based on carboxylated MWCNTs for aflatoxin B_1_ detection. SEM images of the c-MWCNTs/ITO films (**B**) before and (**C**) after anti-AFB_1_ immobilization (inset: high-resolution TEM image of c-MWCNTs). (**D**) Cyclic voltammetric (CV) characterization of (a) c-MWCNTs/ITO, (b) anti-AFB_1_/MWCNTs/ITO, and (c) BSA/anti-AFB_1_/MWCNTs/ITO. (**E**) CV response of a BSA/anti-AFB_1_/MWCNTs/ITO bio-electrode at different concentrations of AFB_1_. Reproduced with permission from [25], Copyright @ 2013 Elsevier B.V (Amsterdam, The Netherlands).

Several research studies have addressed the generation of biomaterial–CNT hybrid systems, protein-linked CNTs, and nucleic acid-functionalized CNTs. The chemical shortening of SWCNTs induced by strong acid treatment leads to the formation of carboxylic and phenolic groups at the nanotube ends [24], thus allowing the covalent immobilization of biomaterials, proteins, and nucleic acids on the surface of the SWCNTs. Recently, an electrochemical biosensor was constructed for the detection of human carcinogen aflatoxin B_1_ using a MWCNT deposited indium-tin oxide (ITO) electrode [25]. Monoclonal aflatoxin B1 antibody (anti-AFB_1_) was covalently bonded with COOH terminal of MWCNTs (c-MWCNTs) via strong amide bond (CO-NH) formation (Figure 3A). After conjugation of anti-AFB1 onto the surface of c-MWCNTs, the film morphology was significantly changed (Figure 3B,C). The immobilization of anti-AFB_1_ led to the formation of nanopores and globular structures on the surface of the c-MWCNT film. The magnitude of current in the case of anti-AFB_1_/MWCNTs/ITO (curve, *b*) decreased to 3.07 × 10^−4^ with peak-to-peak separation of 0.362, indicative of hindered charge transfer owing to insulating nature of antibodies. In the case of BSA/anti-AFB_1_/MWCNTs/ITO, further decrease in the magnitude of current occurred, attributed to the blocked non-specific active sites with BSA (Figure 3D). To investigate the AFB_1_ concentration dependence, the CV response of BSA/anti-AFB_1_/MWCNTs/ITO bio-electrode was monitored by varying the AFB_1_ concentration (Figure 3E). Consequently, the response current increased as the concentration of AFB_1_ increased. This result was attributed to strong affinity between antigens and antibodies, which leads to efficient charge transfer to the electrode surface.

**Figure 4:**
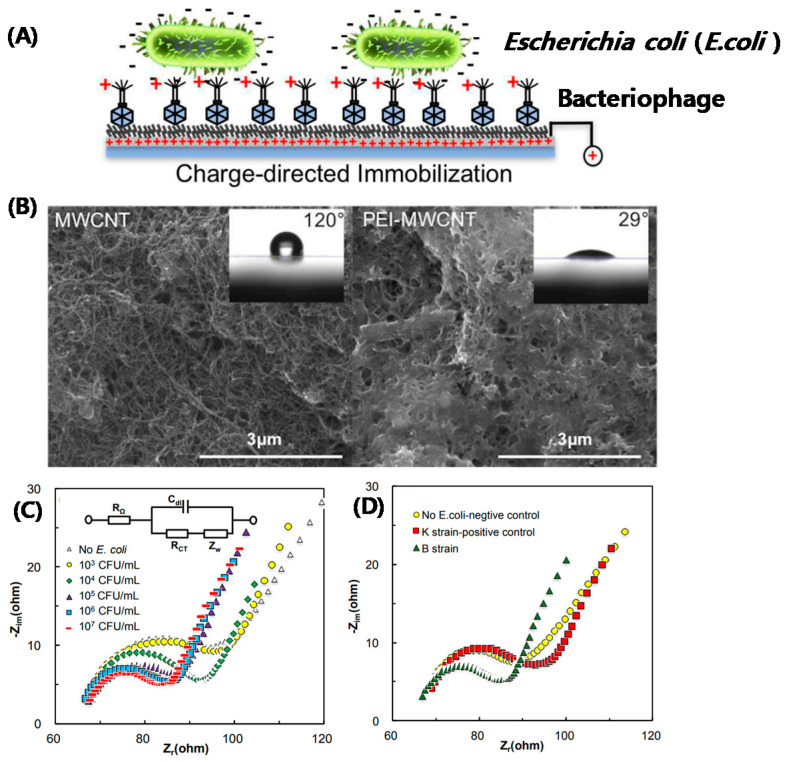
Charge-directed immobilization of T2-bacteriophage on CNT-based electrode of whole-cell electrochemical biosensors. (**A**) Schematic representation of the charge-directed orientation and immobilization of T2-bacteriophage onto PEI-functionalized CNT on an electrode surface. (**B**) SEM images of bare CNTs and PEI-modified CNTs. Inset: photographs of a water droplet on each substrate and their contact angles. (**C**) Nyquist plot of T2-modified electrodes in the absence and presence of *E. coli* B at different concentrations (Inset: Randles equivalent circuit), (**D**) Nyquist plot of T2-modified electrodes in the absence and presence (10^6^ CFU/mL) of native host (B strain) and non-host (K strain) *E. coli*. Reproduced with permission from [30], Copyright @ 2017 American Chemical Society (Washington, DC, USA).

Patolsky et al. reported on the structural alignment of the glucose oxidase (GOx) and flavin adenine dinucleotide (FAD) cofactor on electrodes using SWCNTs as electrical connectors between the enzyme redox centers and electrodes [26]. The surface-assembled GOx was electrically contacted to the electrodes, which acted as conductive nanoneedles that electrically wired the enzyme redox-active site to the transducer surface. Recently, a large derivative of amperometric biosensors based on CNT-modified electrodes have been developed, which can sense a current produced when a potential is applied between two electrodes and can detect electroactive species present in biological samples [11]. Fei et al. reported that platinum (Pt) was electrochemically deposited on the activated CNT/graphite electrode by electrochemical reduction of Pt (IV) complex ions on the surface of CNTs; the cysteine (cySH) on Pt/CNT electrodes was detected by cyclic voltammetry [27]. Zhu et al. reported a bi-enzyme amperometric biosensor for selective and sensitive detection of glucose, in which the enzymes were immobilized on the SWCNT substrate [28]. The bi-enzyme system, consisting of glucose oxidase (GOx) and horseradish peroxidase (HRP), was entrapped in the electropolymerized pyrrole film, and direct electron communication was established by SWCNTs between HRP and the electrode. Liu et al. developed a flow injection amperometric CNT-based glucose biosensor using cationic poly (diallyldimethylammonium chloride) (PDDA). To create this, two layers of PDDA were deposited on the negatively charged surface of CNTs in a layer-by-layer process; the GOx was immobilized onto the CNT surface by alternative assembly of the two PDDA layers with the GOx layer. The sandwich structure of PDDA/GOx/PDDA/CNT/GC formed by ionic interaction provided a favorable condition to maintain the bioactivity of GOx and exhibited excellent sensitivity toward H_2_O_2_ with a detection limit of 7 μM [29]. Yan et al. constructed whole-cell *E. coli* electrochemical biosensors using the negatively charged head of T2 bacteriophage on positively charged MWCNTs functionalized with polyethylenimine (PEI) [30]. To facilitate T2 bacteriophage immobilization onto the surface of CNT-modified electrodes, a positive potential was applied to the working electrode for phage deposition, followed by the covalent linkage of phage through 1-pyrenebutanoic acid succinimidyl ester onto PEI-functionalized CNTs (Figure 4A). The SEM images of CNTs obtained before and after PEI modification distinguish the morphology of the nanostructured electrodes before and after cationic polymer functionalization. The amine groups existing at the surface increased the surface energy of the CNTs and thus made the surface more hydrophilic, resulting in the low contact angle of 29° (see inset in Figure 4B) between a water droplet and a CNT-coated surface (Figure 4B). Figure 4C illustrates the representative open circuit Nyquist plots measured using the phage-modified nanostructured electrodes for the 5 mM Fe(CN)_6_^3−/4−^ redox couple obtained in both the absence and presence of bacteria in the electrolyte solutions of different bacterial concentrations. The equivalent electrical circuit (Randles circuit) used to fit the Nyquist data is shown as an inset in Figure 4C. The capture of bacterial cells on the phage-modified electrode surface altered the surface properties of the electrode, which in turn varied the impedance measured on the biosensor electrode. Figure 4D shows the Nyquist plots of T2 phage-modified electrodes in the strain specific detection of Escherichia coli (*E. coli*) (native host: B strain and non-host: K strains). The absence of variation in the charge-transfer resistance for the K strain compared with the B strain clearly demonstrates that the phage biosensor is highly specific only to the target host (*E.coli* B strain) of the bacterial species.

### 2.3. Optical CNT Biosensors

**Figure 5:**
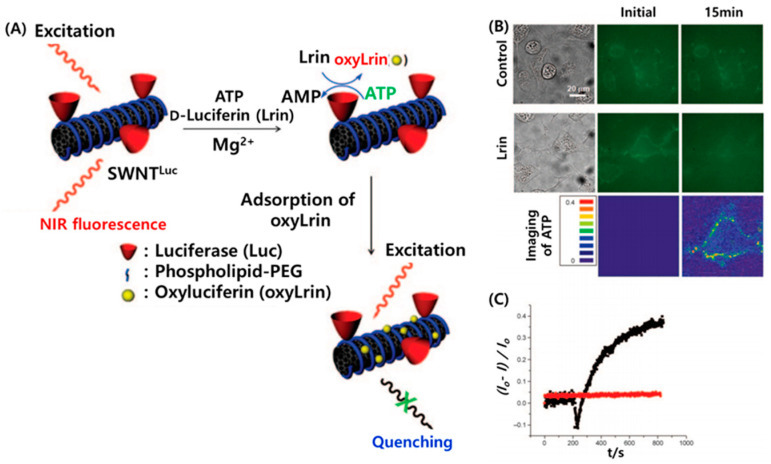
ATP detection in living cells using a SWCNT/luciferase hybrid: (**A**) Schematic description of the SWCNTLuc sensor for cellular ATP detection. The enzymatically oxidized production (oxyLrin) of d-luciferin quenches the NIR fluorescence of SWCNTs. (**B**) NIR fluorescence images of HeLa cells containing the SWCNTLuc sensor with and without addition of Lrin. The NIR fluorescence in HeLa cells is quenched by the addition of Lrin (240 μM), which is dependent on the ATP concentration. (**C**) Real-time and quantitative tracking of NIR fluorescence of the SWCNTLuc sensor responding to ATP in HeLa cells with (black trace) and without (red trace) addition of Lrin. Reproduced with permission from [35], Copyright @ 2010 WILEY-VCH Verlag GmbH & Co. KGaA, Weinheim (Weinheim, Germany).

The exceptionally unique optical properties of CNTs encouraged extensive research for developing optical biosensors for biomarker detection and imaging. The basic principle of optical biosensors is to detect the interaction between the target biomolecules and analytes by measuring the emission of light (UV, visible, infrared, or fluorescence) rather than electrons. [31]. Some studies focused on SWCNT fluorescence emission signals (wavelength and intensity), which were significantly affected by surface functionalization, environmental changes, or interactions with target biomolecules, thereby making SWCNTs well suited for fluorescence-based sensing applications [32,33]. Likewise, non-covalent assembly of SWCNTs with DNA containing aptazyme-labeled detection probes yielded an optical biosensor of single-stranded DNA (ssDNA); this self-assembled DNAzyme acted as a catalyst for the generation of chemiluminescence through the oxidation of luminol by H_2_O_2_, which resulted in an amplification of the detection signal [34]. Kim et al. reported a biosensor selective to adenosine 50-triphosphate (ATP), but not to adenosine 5′-monophosphate (AMP), adenosine 5′-diphosphate (ADP), cytidine 5′-triphosphate (CTP), or guanosine 5′-triphosphate (GTP), which was achieved by conjugating SWCNTs to luciferase (SWCNT^Luc^) in living HeLa cells (Figure 5A) [35]. Figure 5B shows the NIR fluorescence images of HeLa cells containing the SWCNT^Luc^ sensor without Lrin (Figure 5B, top) and with the addition of Lrin (Figure 5B, center). The NIR fluorescence in HeLa cells was quenched after the addition of Lrin (240 μM), which correlated to the ATP concentration. The fluorescence quenching for ATP detection in the cells is clearly observed in the quantitative and real-time tracking of NIR fluorescence (Figure 5C).

The Heller group engineered an optical sensor for alkylating agents and reactive oxygen species (ROS) for the distinct response of genotoxin immobilized on the CNT surface, which measured alkylating and ROS activity within the living cells through NIR photoluminescence [36]. The SWCNT near-infrared (NIR) fluorescence was created by generation of a redox-quenching intermediate from the target analyte, then spatial and temporal information of NIR detection in ATP living cells was achieved with a detection limit of 240 nM.

## 3. Carbon Nanodiamonds (NDs)

Nanodiamonds (NDs) are nanosized (5–100 nm) carbon structures composed of sp^3^-hybridized carbon atoms. Their unique optical and electronic characteristics allow them to be used in specific biosensing applications. Among CNs, NDs are exceptional due to their biocompatibility, low toxicity, thermal conductivity, refractive index, and the chemical inertness of their diamond core, with a highly diverse surface containing functional groups including hydroxyl, carbonyl, ether, and carboxyl groups [37,38]. As a delivery cargo of target molecules, NDs can serve as a useful platform with semi-octahedral carbon structures with a number of physical properties, such as charged surface facets and chemical functional groups formed via covalent or non-covalent mechanisms on their surface. The covalent methods can generate stable ND surfaces, but require complex processes. Meanwhile, the non-covalent modifications that utilize a simple procedure are widely employed for the functionalization of ND surfaces.

### 3.1. Synthesis and Purification

**Figure 6:**
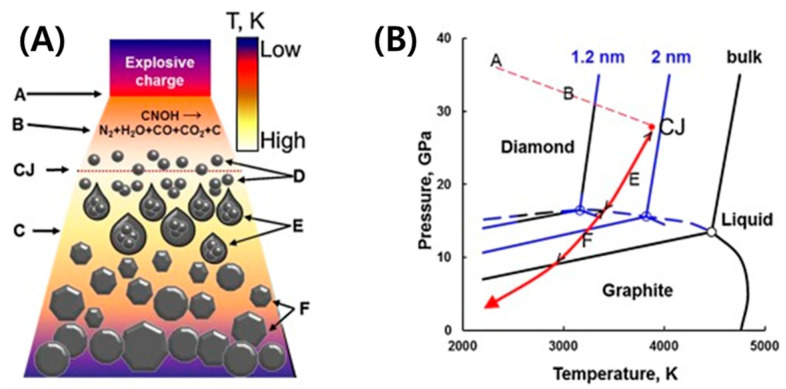
(**A**) Schematic of the detonation wave propagation showing: **A** the front of the shock wave caused by the explosion; **B** the zone of the chemical reaction in which the explosive molecules decompose; (CJ) the Chapman–Jouguet plane (where pressure and temperature correspond to point CJ in (**B**), indicating the conditions when the reaction and energy release are essentially complete); C the expanding detonation products; D the formation of carbon nanoclusters; E the coagulation into liquid nanodroplets; and F the crystallization, growth, and agglomeration of nanodiamonds. Reproduced with permission from [43], Copyright @ 2017 Elsevier Inc (Amsterdam, The Netherlands).

Commercially, there are several detonation techniques available for nanodiamond production. The three most common procedures used for producing NDs are laser ablation [39], high-energy ball milling of high-pressure, high-temperature diamond microcrystals [40], and plasma-assisted CVD [41]. Diamond nanoparticles have been classified by ND size into nanocrystalline particles (10–100 nm), which are produced by shock-wave compression of graphite or detonation of a carbon/explosives mixture, ultrananocrystalline NDs (2–10 nm) produced by detonation or laser ablation, and the smallest category, diamondoids (1–2 nm), produced by petroleum processing with hydrogen termination [42]. NDs can be created from an explosive mixture having an overall negative oxygen balance, which provides both a source of carbon and energy for the conversion (Figure 6A). The detonation wave propagation is illustrated in Figure 6A. At Stage A, the explosion to generate the shock wave is initiated, then the chemical reaction that causes decomposition of the explosive molecules occurs at Stage B. Stage CJ is the Chapman–Jouguet plane indicating the conditions when the reaction and energy release are essentially complete, in which pressure and temperature correspond to point A in Figure 6B. The detonation products are expanded at high temperature in Stage C and form carbon nanoclusters in Stage D. The liquid nanodroplets are coagulated in Stage E. Finally, the crystallization, growth, and agglomeration of nanodiamonds occur in Stage F [43].

The detonation for the creation of NDs takes place in a closed chamber filled with an inert gas (dry synthesis) or water (wet synthesis) coolant. The detonation soot contains diamonds with a diameter of 4–5 nm up to 75 wt% along with other carbon allotropes and impurities [44]. The ND formation during detonation increases instantaneously at appropriate pressures and temperatures of the Jouguet point (point CJ in Figure 6B), which are high enough to produce liquid carbon at the nanoscale. As the temperature and pressure decrease along the isentrope (red line), carbon atoms condense into nanoclusters, which further coalesce into larger liquid droplets and crystallize.

For purification of NDs with a perfect crystalline structure, the non-diamond carbon can be oxidized by air or ozone-enriched air at elevated temperatures [45,46]. Oxidation by air is a robust, cost-effective purification technique, capable of increasing the diamond content from ~25 wt% to > 95 wt% [37]. First, the oxidation technique removes various functional groups existing on the ND surface and produces oxygen-containing surface species, converting various grades of ND powders into a single material. The ozone-purified technique can produce smaller-sized NDs in aqueous dispersions (~160–180 nm) and a substantially higher content of faceted 3–5 nm particles, compared to acid purification. Surface reduction in a hydrogen atmosphere has also been attempted for purification of NDs; however, non-diamond carbons are not completely removed in this method [47].

### 3.2. Nanodiamonds for Biosensing Applications

**Figure 7:**
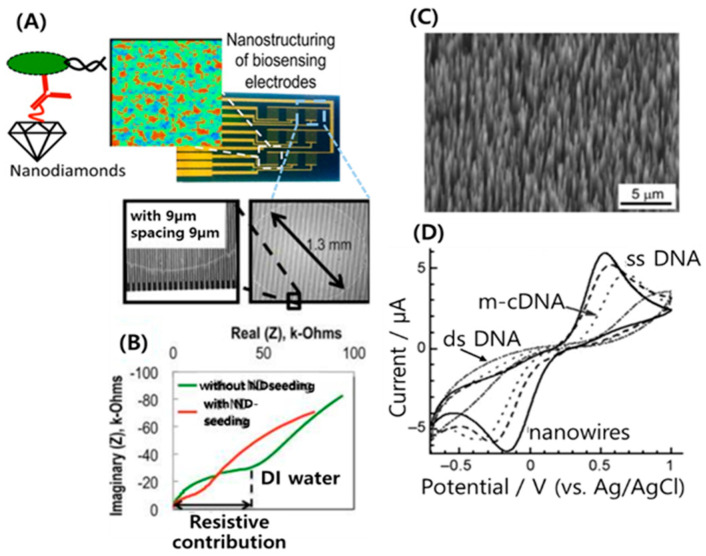
(**A**) Illustrated interaction of hydrogenated NDs/antibody/bacteria and optical images of a biosensor chip containing an array of nine interdigitated electrode (IDE) pairs that were fabricated to demonstrate the application of ND seeding layer for chemically stable covalent linkage of antibodies to electrodes. (**B**) Representative plot of real vs. imaginary part of the impedance measured in deionized water on an IDE before and after ND seeding. Reproduced with permission from Ref. [48], Copyright @ 2014 American Chemical Society (Washington, DC, USA). The SEM image (**C**) of vertically aligned and boron doped diamond nanowires. (**D**) Cyclic voltammograms of 1.0 mM [Fe(CN)_6_]^3−/4−^ in pH 7.4 phosphate buffer on nanowires before (—) and after functionalization with ss-DNA (- - - -), single-base-mismatched DNA (m-cDNA) (••••), and ds-DNA (•–•–). The scan rate was 100 mV s^−1^. Reproduced with permission from [49], Copyright @ 2008 WILEY-VCH Verlag GmbH & Co. KGaA, Weinheim (Weinheim, Germany).

Zhang et al. [48] reported that aligned NDs can be sufficiently hydrogenated to conjugate with antibodies using UV-alkene chemistry, and the higher sensing of *E. coli* O157:H7 can be obtained at a concentration of 10^6^ cfu/mL. The array of 3 × 3 interdigitated electrodes (IDEs) for seeding with NDs were fabricated using 200 nm Au contacts with 25 nm Cr as the adhesion layer, where each finger is 9 μm thick with 9 μm spacing (Figure 7A). Impedance spectra of IDEs in deionized water (as shown in Figure 7B) show that the resistive or charge-transfer contribution to the overall impedance decreases with ND seeding. This implies that the ND seeds form electrically conductive islands between the electrode fingers.

Yang et al. introduced the electrochemical application of vertically aligned diamond nanowires (NWs) for DNA sensing [49]. These metal-like NWs were fabricated from boron-doped single-crystalline CVD diamond using of diamond nanoparticles as a hard mask. This group produced wires that were 3–10 nm long and spaced 11 nm apart (Figure 7C). NWs separated by approximately 11 nm were selected because anchoring DNA molecules onto these wires would result in a density of DNA of about 10^12^/cm^2^ with a high efficiency of DNA sensing. The tips of the nanowires were functionalized electrochemically with phenyl groups used to conjugate the oligonucleotide molecules to diamond [49]. [Fe(CN)_6_]^3−/4−^ redox mediators have been used for the investigation of DNA sensing on diamond-based biosensors. Figure 7D shows a cyclic voltammogram after exposure to single-base-mismatched DNA(m-cDNA). The amplitude was decreased by about 20% relative to ssDNA owing to nonintentional bonding. However, clear discrimination between complementary ds-DNA and single-base-mismatched DNA bonding was detected. These results clearly demonstrate that [Fe(CN)_6_]^3−/4−^ is a good indicator for DNA bonding to diamond.

Matrix metalloproteinases (MMP) proteins are a family of zinc-containing endopeptidases that cleave specific peptide sequences [50]. Xin et al. quantified MMP9 activity through cleavage detection using a pair of Förster resonance energy transfer (FRET) molecules including fluorescein isothiocyanate (FITC) molecules and 5-carboxytetramethylrhodamine (5-TAMRA), which were placed on either side of the peptide. Energy transfer between FITC and 5-TAMRA occurred when the peptide was uncleaved, resulting in fluorescence quenching of the FITC molecule. Meanwhile, the FITC emission signal was measured, which indicated no energy transfer to 5-TAMRA when MMP9 cleaved the peptide, although the distance between FITC and 5-TAMRA increased [51].

Hamers et al. demonstrated a label-free biosensor based on nanocrystalline diamond films with frequency-dependent interfacial electrical properties, which were covalently linked to DNA oligonucleotides. The DNA molecules induced a field effect in the diamond space-charge layer, altering the impedance of the diamond films [52].

**Figure 8:**
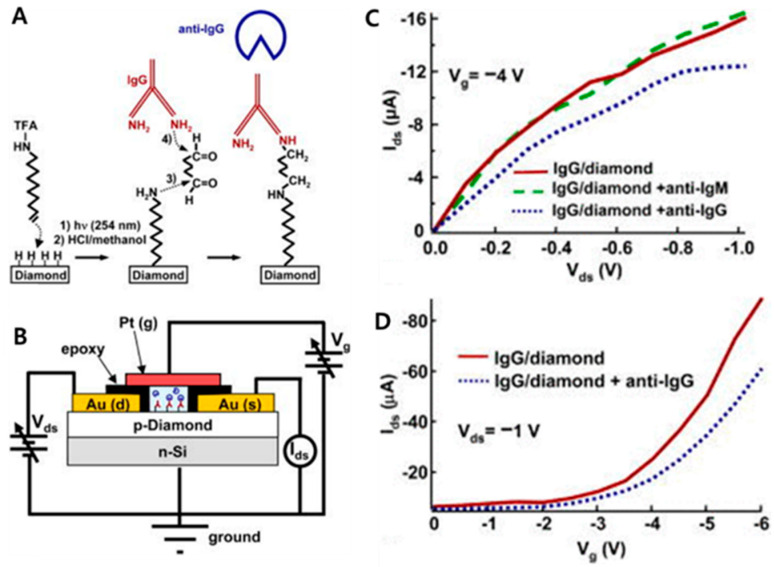
(**A**) Schematic illustration for procedures of linking human IgG to diamond surfaces. (**B**) Schematic illustration of a diamond-based bio-FET structure. (**C**) Ids–Vds curves at Vg = −4 V for an IgG-modified diamond FET before (solid line) and after exposure to anti-IgM (dashed line) and to anti-IgG (dotted line). (**D**) Ids–Vg curves at Vds = −1 V for IgG-modified diamond FET before (solid line) and after exposure to anti-IgG (dotted line). Reproduced with permission from Ref. [53] Copyright @ 2004 American Institute of Physics (College Park, MD, USA).

Yang et al. [53] reported a biologically sensitive field-effect transistor (Bio-FET) using an ND thin film by linking human immunoglobulin G (IgG) to the diamond surface. Undoped nanocrystalline diamond thin films (p-diamond) were grown on n-type silicon (10 Ωcm) substrates by microwave-enhanced plasma CVD (Figure 8B). The human antibody (i.e., IgG) was attached to the diamond surfaces via a multi-step process for the capability of biomolecular recognition (Figure 8A). The diamond surfaces were hydrogenated using a radio-frequency plasma, and then covalently linked (step 1) with an organic monolayer film that generated protected amine groups (i.e., 10-aminedec-1-ene protected with trifluoracetic acid) to the surface through photoexcitation at 254 nm. Some exposed aldehyde groups were created on the surface of diamond via de-protection in 0.36 M HCl/methanol (step 2) followed by a reaction with 3% glutaraldehyde solution in a sodium cyanoborohydride coupling buffer for 2 h (step 3). Subsequently, Au was sputtered and coated on the surface of aldehyde-terminated diamond. The coated Au electrodes were then covered with a thin epoxy layer to protect them from the solution (Figure 8B). Finally, the diamond with an aldehyde-terminated surface was immersed in a 1 mg/mL IgG solution for approximately 8 h (step 4). To stabilize unreacted aldehyde groups, a further reaction was performed by immersion in a solution of 0.1 M glycine in sodium cyanoborohydride buffer. In Figure 8C, the solid line indicates the drain current versus drain-source voltage for an IgG-modified diamond FET at a gate-source voltage of −4 V. After exposure to anti-immunoglobulin (anti-IgM) as a control, the response (green dashed line) in the drain current remained unchanged. However, the response (blue dotted line) became pronouncedly decreased after exposure to anti-IgG. In addition, the biomolecular recognition between the IgG-modified surface and the solution-phase anti-IgG was detected via the changes in drain current as a function of the gate voltage (Figure 8D). Consequently, the electrical measurements proved that the bio-FET device composed of an IgG-modified diamond exhibits a response specific to the anti-IgG antibody.

Tatsuma et al. [54] examined the direct electron transfers that occur from boron-doped diamond (BDD) electrodes to heme undecapeptide and HRP, and evaluated their application to H_2_O_2_ biosensors. As-grown and oxygen-plasma-treated diamond electrodes on which heme peptide was adsorbed exhibited cathodic current responses to H_2_O_2_ based on the direct electron transfer. The electron transfers to compounds I and II of HRP from the diamond electrodes were much slower compared to that from an edge-oriented pyrolytic graphite or glassy carbon, indicative of the critical role of the π electrons of an sp^2^ carbon in the direct electron transfer to the heme moiety of HRP. Conversely, the diamond electrode exhibited significantly selective sensitivity to the heme moiety of HRP.

Notsu et al. improved BDD electrodes that were covalently linked with tyrosinase for the detection of phenol derivatives. BDD was anodically polarized for the introduction of hydroxyl groups onto its surface, then treated with (3-aminopropyl) triethoxysilane, and finally was coated with a tyrosinase film cross-linked with glutaraldehyde. The modified electrodes responded amperometrically to phenol derivatives including estrogenic derivatives. Interference from direct reduction of oxygen at the electrode surface was almost negligible; the overpotential for the oxygen reduction at BDD was greater than those for most conventional electrode materials [55].

## 4. Graphene (GR) for Electrochemical Biosensors

Graphene (GR), a single layer of graphite, is a crystalline allotrope of carbon in the form of a single layer of atoms. Graphite is comprised of layers of sp^2^-bonded carbon atoms arranged in a hexagonal lattice, in which graphene sheets are held together in place by weak van der Waals forces. Owing to GR’s exceptional optical, electronic, and magnetic properties, its use enables a simple rapid, low-cost biosensing platform. Further, it is a two-dimensional semiconductor material with a zero band gap and exhibits a strong ambipolar electric field effect. The mobility of electrons within the GR layers is one hundred times higher than electron mobility in silicon [56]. Furthermore, the GR-based FETs have been used as label-free biosensors due to higher sensitivity, easier operation, and simpler sample preparation processes compared to the biological sensors with dye-labeled biomolecules [57,58,59].

### 4.1. Graphene Synthesis

#### 4.1.1. Exfoliation

Exfoliation techniques are widely used to create high-quality graphene using mechanical or chemical energy to break weak van der Waals bonds and separate out individual graphene sheets. Dry etching in oxygen plasma was the first technique to create many 5 µm deep mesas (with surface area 0.4 to 4 mm^2^) from highly oriented pyrolytic graphite sheets 1 mm thick [60]. The mesas were then subjected to a baking process to adhere them to the photoresist, and subsequently a scotch tape was used to peel off layers from the graphite sheet. Thin flakes, attached to the photoresist, released in acetone and transferred to a Si substrate, were found to contain graphene sheets varying from a single layer to a few layers in thickness [61]. Stankovich et al. [62] suggested a slightly different approach of exfoliation using the hydrophobicity of graphite oxide (GO) and exfoliated GO nanosheets, in which these materials underwent ultrasonication in an aqueous suspension followed by the attempted reduction of the films in hydrazine hydrate at 100 °C for 24 h. The liquid phase-based exfoliation enabled the production of large-size graphene [63]. However, the structure of the liquid phase exfoliated products suffered from several defects, due to oxidation and reduction processes, leading to considerably poor electrical properties of graphene.

#### 4.1.2. Thermal Chemical Vapor Deposition (CVD) Techniques

Thermal CVD for the synthesis of planar few-layer graphene was developed in 2006. Somani et al. reported a CVD technique based on a natural, eco-friendly, low-cost camphor as a potential source of raw carbon for the production of graphene on Ni foils [64]. Camphor was first evaporated at 180 °C and then pyrolyzed in another chamber of the CVD furnace at 700 to 850 °C using argon as a carrier gas. Few-layer graphene sheets were ultimately observed on the surface of the Ni foils. Approximately 35 layers of graphene sheets were generated by the CVD technique. Obraztsov [65] et al. used a gas mixture of H_2_ and CH_4_ (92:8 ratio) as a precursor for graphene synthesis; the precursor gas mixture was activated by DC discharge under a total gas pressure of 80 torr. Yu et al. [66] identified a process for graphene synthesis using a precursor gas mixture of CH_4_, H_2_, and Ar (0.15:1:2 ratio) at a total flow rate of 315 cm^3^/min at STP. After understanding the mechanism of graphene growth, Wang et al. [67] proposed a new method of growing substrate-free few-layered graphene sheets. In their graphene synthesis process, MgO-supported Co catalysts were used to grow graphene in a ceramic boat at 1000 °C for 30 min under an atmospheric gas envelope of CH_4_ and Ar (1:4 volume ratio). Further washing was implemented by using concentrated HCl to remove MgO and Co, followed by washing with distilled water several times and drying at 70 °C.

**Figure 9:**
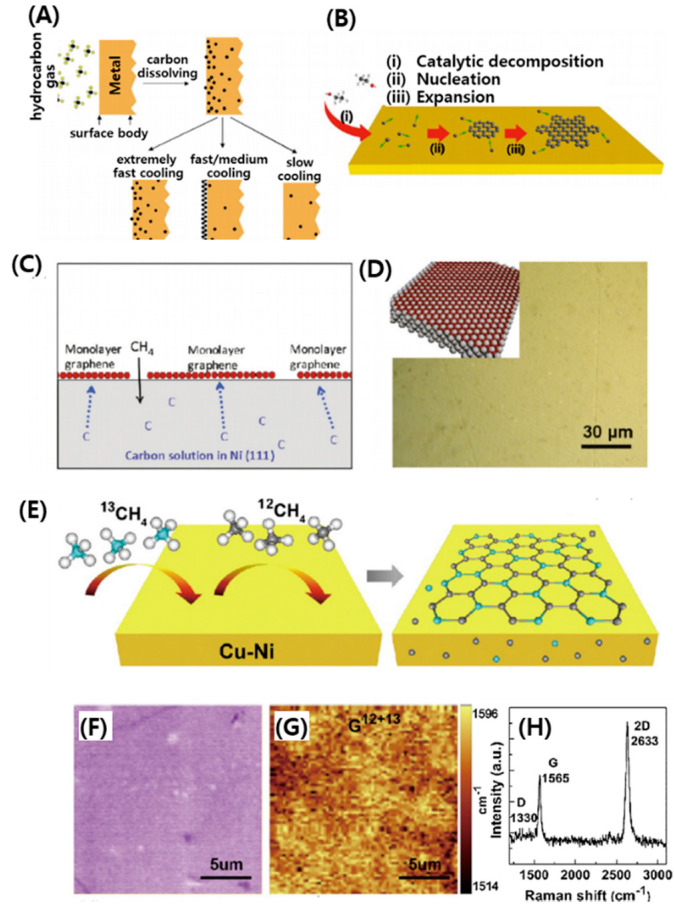
Schematics of CVD graphene grown on (**A**) Ni and (**B**) Cu foil. (**C**) Schematic diagrams of graphene growth mechanism on Ni (111) and (**D**) the optical image of graphene growing on Ni (111) surfaces after the CVD process. (**E**) Schematic diagrams of the growth process of carbon isotope labeled graphene, and the possible distribution of ^12^C and ^13^C atoms in graphene films and inside the Cu–Ni alloy. (**F**) Optical micrograph of a monolayer graphene transferred onto a 285 nm SiO_2_/Si substrate and (**G**) the corresponding Raman map generated by the position of the G band. (**H**) A Raman spectrum collected from the corresponding monolayer graphene. Reproduced with permission from [66], Copyright @ 2008 American Institute of Physics and [71,72], Copyright @ 2010, 2012 American Chemical Society (Washington, DC, USA).

Recent progress on the synthesis of large-area monolayer graphene on metals is comparatively mature. When metals (e.g., Ni and Fe) with a high carbon solubility are used as a substrate, the carbon diffuses into the heated substrate according to the solubility of carbon [68]. After cooling the substrate, the dissolved carbon precipitates and forms graphene sheets on its surface [66,69]. This “segregation” process is depicted clearly in Figure 9A [66]. For metals such as Cu with low carbon solubility, carbon atoms tend to nucleate and laterally expand around the core nucleus to create graphene domains through the decomposition of a hydrocarbon, which is catalyzed by the substrates at high temperature (Figure 9B) [70]. Specifically, Figure 9C,D show, respectively, a schematic diagram and optical image of the growth mechanism of graphene during the carbon segregation and precipitation on Ni (111). The surface of Ni (111) is very smooth and without grain boundaries, and allows uniform segregation of carbon onto the Ni (111) surface, resulting in the formation of single-layer graphene [71]. In addition to pure metals, metal alloys have been utilized for the generation of graphene layers. Interestingly, bilayer graphene was generated on Cu-Ni alloy foils [72]. Typically, carbon isotopes (i.e., ^12^C and ^13^C) were introduced into the heated alloy foils to form bilayer graphene on the foils’ surfaces (Figure 9E). Figure 9F,G show an optical micrograph and a Raman map of the G peak position of a monolayer graphene transferred onto a SiO_2_/Si substrate. The uniform contrast of the entire Raman map without distinguishable isotopic separation suggests that ^12^C and ^13^C were randomly distributed over the graphene film without local segregation. The D, G, and 2D bands located at the frequencies of 1330, 1565, and 2633 cm^−1^, respectively, indicate that the graphene film was composed of a mixture of 70% ^12^C and 30% ^13^C (Figure 9H).

#### 4.1.3. Plasma Enhanced Chemical Vapor Deposition (PECVD) Techniques

PECVD is a deposition method that enables the deposition of graphene films by converting a precursor from a gas state to a solid state on substrates at a lower temperature than that of standard CVD. The PECVD method could expand the variety of applications of graphene due to its versatility of producing graphene on any substrate, its ability to control the thickness of graphene layers, and its capacity for large-scale production. For instance, Obraztsov et al. proposed a PECVD method that is performed under direct current discharge to produce nanostructured graphite-like carbon [73]. In the process, Si wafers and Ni, W, Mo, and certain other metal sheets were used as substrates, and a gas mixture composed of CH_4_ and H_2_ was introduced at a total gas pressure of 10 to 150 torr [74]. In another method, a radio-frequency PECVD approach was used to generate graphene on a variety of substrates (e.g., Si, W, Mo, Zr, Ti, Hf, Nb, Ta, Cr, 304 stainless steel, SiO_2_, and Al_2_O_3_) without any special surface treatment or catalyst deposition [75].

### 4.2. Enzymatic Glucose Biosensor Based on Graphene

GR nanocomposites with inorganic nanostructures [76,77,78,79], conducting polymers [80,81,82,83] and organic materials [84] can enhance the analytical response of sensors and biosensors, mainly improving their sensitivity, limit of detection, and reproducibility of electrochemical biosensors for the detection of biomolecules. Functionalized GR/reduced GO (rGO) with a variety of materials, iridium oxide [85], Au nanostructures [86,87,88,89], AgNP [90,91], FeNP [92], PtNPs [93,94,95,96], Pd–Pt NPs [97,98], TiO_2_ NPs [99,100], CuO_2_ NPs [101], ZrO_2_ NPs [102], or CNTs [103], have been used as electrochemical biosensors for enhancing the electron transfer property between electrode substrates and enzymes.

**Figure 10:**
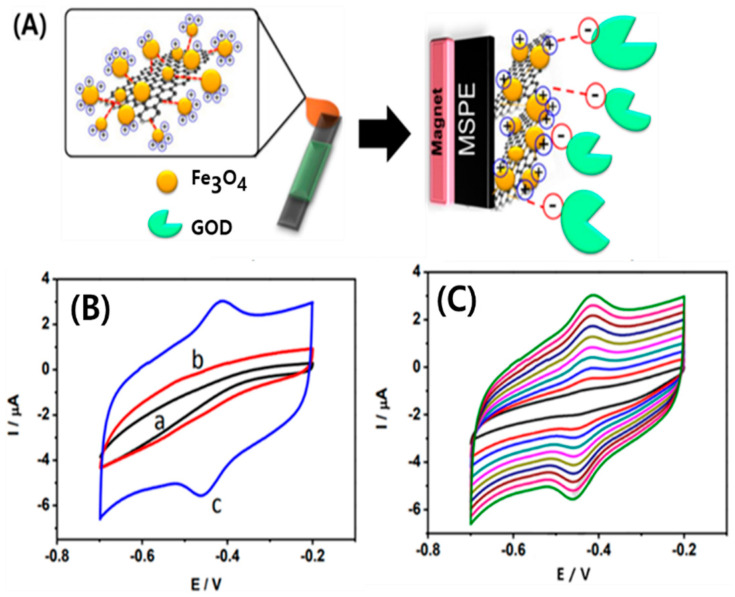
Self-assembly of glucose oxidase (GOD) on rGO-magnetic nanoparticles nanocomposite-based direct electrochemistry for reagentless glucose biosensor. (**A**) Schematic illustration of rGO-Fe_3_O_4_/GOD modified MSPE. (**B**) Cyclic voltammograms of the different modified electrodes with (a) GOD/MSPE, (b) Fe_3_O_4_/GOD/MSPE, and (c) rGO-Fe_3_O_4_/GOD/MSPE, in 0.1 M PBS at pH 7.0 with N_2_-saturated at a scan rate of 100 mV/s. (**C**) CVs of the rGO-Fe_3_O_4_/GOD/MSPE in 0.1 M PBS at pH 7.0 at different scan rates, inner to outer: 10, 20, 30, 40, 50, 60, 70, 80, and 100 mV/s. Reproduced with permission from [104], Copyright @ 2017 Elsevier B.V (Amsterdam, The Netherlands).

Pakapongpan et al. [104] developed a novel approach for the immobilizadtion of a highly selective and stable glucose biosensor, based on direct electrochemistry by self-assembly of glucose oxidase (GOD) on rGO; this was covalently conjugated to magnetic nanoparticles (Fe_3_O_4_ NPs) modified on a magnetic screen-printed electrode (MSPE) (Figure 10A). This GR-based biosensor showed a fast amperometric response to glucose with a wide linear range from 0.05 to 1 mM and good sensitivity (5.9 μA/mM). As shown in Figure 10B, the cyclic voltammograms (CVs) of different GOD-modified electrodes were investigated in the N_2_-saturated PBS at a scan rate of 100 mV/s. No redox peaks were observed for either the GOD/MSPE (curve a) or the Fe_3_O_4_-GOD/MSPE (curve b). Meanwhile, a well-defined redox peak with anodic and cathodic peak potentials (E_pa_ and E_pc_, respectively) at −0.418 V and −0.452 V, respectively, was observed in curve c. The CV performance at the rGO-Fe_3_O_4_/GOD-modified MSPE was influenced by scan rate as shown in Figure 10C. During the redox processes of the nanocomposite, primarily symmetric anodic and cathodic peaks clearly appeared at relatively slow scan rates. Linear increases in the anodic and cathodic peak currents (*I*_pa_ and *I*_pc_, respectively) were observed upon increasing the scan rate from 10 to 100 mV/s, which indicates that the redox reaction of the GOD on the rGO-Fe_3_O_4_-modified electrode was a quasi-reversible surface-controlled process.

Park et al. [105] reported a new platform based on rGO-carboxylated polypyrrole (C-PPy) nanotube (NT) hybrids as a conducting channel for sensitive and rapid response of a FET sensor with glucose. Glucose oxidase (GOx) was immobilized onto the rGO/C-PPy NTs hybrid biosensor via a chemical coupling reaction. Glucose detection was enabled by GOx, which catalyzes the oxidation of glucose; the detection was achieved in a rapid response with high sensitivity to glucose with a detection limit of 1 nM.

Qi et al. [106] successfully constructed a glucose sensor based on AuNP-decorated GO nanocomposites (GO-Ph-AuNP) via aryldiazonium salt chemistry for loading GOx, which is capable of monitoring glucose consumption in live cells. The direct electrochemistry of GOx was successfully realized on GO-Ph-AuNP-modified glassy carbon electrodes with a heterogeneous electron transfer rate constant of 8.3 s^−1^. The GOx immobilized on the GO-Ph-AuNP electrode retained good electrocatalytic activity toward glucose over a linear concentration range from 0.3 to 20 mM with a sensitivity of 42 μA∙mM^−1^∙cm^−2^.

Rabti et al. [107] reported a new biosensor platform based on a nanoporous flat electrode that was fabricated via drop coating a nanocomposite solution consisting of rGO and chitosan onto a polyester substrate. The resulting biosensor yielded a linear response to glucose concentration with an improved detection limit and sensitivity in the measurement of artificial saliva. Rafighi and collaborators [108] reported a novel approach for the fabrication of a glucose biosensor based on the immobilization of GOx on a graphene poly ethyleneimine-gold nanoparticles hybrid (GNS-PEI-AuNPs) using glutaraldehyde as cross-linking reagent. The fabricated glucose biosensor linearly responded toward glucose in the concentration range between 1 and 100 μM with a sensitivity of about 93 μA∙mM^−1^∙cm^−2^.

**Figure 11:**
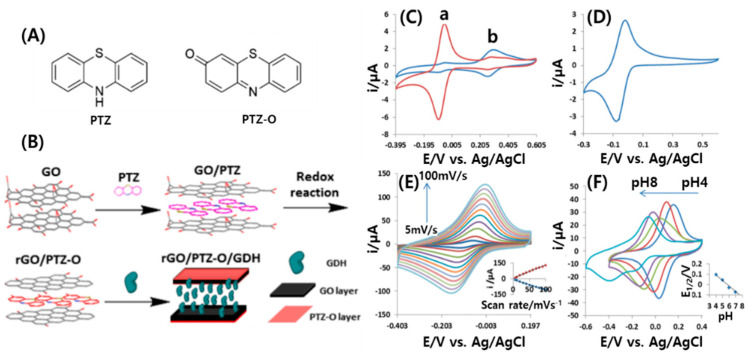
(**A**) Chemical structures of phenothiazine (PTZ) and 3H-phenothiazine-3-one (PTZ-O). (**B**) Schematic representation of the procedure for preparing the rGO-based bio-composite. Cyclic voltammograms (CVs) of GC electrodes: (**C**) (a) rGO/PTZ after oxidation and (b) PTZ in solution; (**D**) pure PTZ-O extracted from rGO; (**E**) different scan rates. Inset: scan rate-dependent peak currents of rGO/PTZ-O/GCE; (**F**) rGO/PTZ-O/GCE pH dependence; inset: middle point potentials vs. pH. Reproduced with permission from [109], Copyright @ 2015 American Chemical Society (Washington, DC, USA).

Ravenna et al. [109] developed a bio-composite material for use in the encapsulation of redox enzymes. The structures of phenothiazine (PTZ) and phenothiazine-O (PTZ-O) were determined by MS and NMR analyses (Figure 11A). The bio-composite material was prepared by modification of rGO films with PTZ-O, in combination with a simple and nondestructive entrapment of glucose dehydrogenase (GDH) within the rGO/PTZ-O films (Figure 11B).

Figure 11C shows a cyclic voltammograms (CV) of rGO modified with PTZ-O (rGO/PTZ-O) and a solution with the soluble fraction of mostly insoluble PTZ using a glassy carbon electrode (GCE) as the working electrode. For the purified PTZ-O extracted from the PTZ-modified rGO matrix, a reversible electrochemical behavior was observed with a middle point potential of E_1/2_ = −70 mV (Figure 11D) and a peak-to peak separation of ca. ΔE = 60 mV, which corresponds to a complete reversible behavior of a one-electron redox process. PTZ-O adsorbed on rGO shows reversible electrochemical voltammograms with a surface-confined process dependence as indicated by the measurements shown in Figure 11E (inset), with a linear dependence of anodic and cathodic peak currents on scan rate. In order to determine the number of electrons transferred per molecule, the voltammetric response of the modified electrode was tested under pH values varying between pH 4.0 to 8.0 (Figure 11F). The formal potential was pH-dependent with a negative shift with increasing pH (inset of Figure 11F). A reduced graphene oxide film with adsorbed phenothiazone was used as a highly efficient composite for electron transfer between flavin adenine dinucleotide (FAD)-dependent glucose dehydrogenase and electrodes.

### 4.3. Non-Enzymatic Glucose Graphene-Based Biosensor

Owing to their large surface-to-volume ratio and ordered arrangement, there has lately been significant interest in poly-dimensional nanostructures in the fabrication of biosensors. The multidimensionality of sensor-active materials is considered to be the benefit for high electroactivity, namely fast electrocatalytic responses toward glucose oxidation. Recently, researchers have reported and fabricated several non-enzymatic biosensors using the hybrids of graphene-multidimensional nanomaterials.

For the detection of glucose via an electrochemical oxidation, Benjamin et al. [110] developed a non-enzymatic biosensor based on an ionic liquid tagged cobalt-salophen metal complex (Co-salophen-IL) immobilized on electrochemically reduced graphene oxide (ERGO). Notably, the Co-salophen-IL/ERGO/SPE biosensor exhibited excellent electrocatalytic activity toward glucose oxidation in the presence of 0.1 M NaOH; the modified electrode showed prominent performance of glucose detection over a wide linear range from 0.2 µM to 1.8 mM.

Mazaheri et al. [111] proposed a hybrid electrode with a novel three-dimensional nanostructure based on rGO/Ni/ZO for the detection of glucose oxidation. The prepared biosensor possessed a rapid electrocatalytic response toward glucose oxidation due to its large surface area and high electroactivity.

An additional novel, stable, and sensitive non-enzymatic glucose biosensor, which was based on the hybrid of bimetallic hollow Ag/Pt nanoparticles-reduced graphene oxide (HAg/PtNPs-rGO), was prepared by a galvanic replacement reaction and the thermal reduction of GO [112]. In this procedure, thermal reduction was highly effective in producing graphene-like films that can render a stable substrate for hollow Ag/Pt nanoparticles.

Gao et al. [113] employed Ni(OH)_2_-ERGO–MWCNT nanocomposites to fabricate a non-enzymatic glucose sensor. GO sheets can serve as surfactants for the stable aqueous dispersion of pristine MWCNTs. In the non-enzymatic graphene-based glucose sensor, MWCNTs acted as conducting bridges between ERGO sheets to enhance the electron transfer rate in the substrate. By combining the advantages of ERGO and MWCNTs with the electrocatalytic capability of Ni(OH)_2_ nanoparticles, the well-designed nanocomposites exhibited excellent sensing performance toward glucose and H_2_O_2_.

## 5. Fullerenes for Electrochemical Biosensors

**Figure 12:**
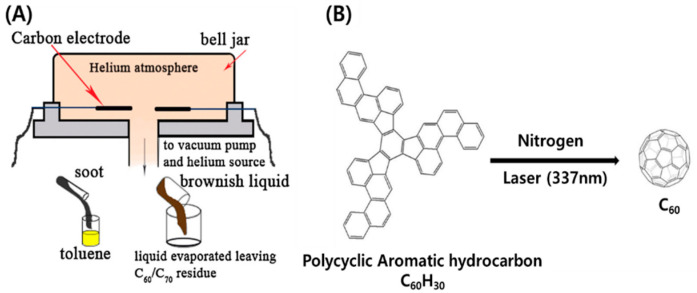
Schematic of fullerene synthesis methods: (**A**) Huffman–Krätschmer method based on evaporation of graphite. Reproduced with permission from [126] Copyright © 2020 Elsevier Ltd. (**B**) Synthetic route to C_60_H_30_ (PAH) and its laser-induced conversion into C_60_. Reproduced with permission from [128], Copyright @ 2001 The American Association for the Advancement of Science.

The unique spherical structure and exceptional physicochemical properties of fullerenes make them very attractive as mediators for various biosensing applications, allowing operation at lower potentials. Hence, it is possible to detect electroactive components while reducing the interferences from the intrinsic electroactive compounds. These hollow carbon spheres possess an excellent electron acceptor capability for efficient electron transfer due to abundant conjugated π electrons [114,115,116]. In addition, they can react with amines and be successfully functionalized with biological moieties. The function of fullerene at the interface between the recognition site and the electrode is depicted in Figure 12A [117]. As an electron mediator between the recognition site and the electrode in electrochemical biosensors, fullerenes significantly enhanced the rate of electron transfer that occurred through the biocatalytic or biochemical reaction of the analytes in contact with the biological elements at the recognition sites. In general, biomaterials such as enzymes, antibodies, organelles, microorganisms, and tissues are utilized as recognition sites in biosensors. Further, fullerene is able to act as an electron acceptor with a dual nature of electrophilic and nucleophilic characteristics accompanying the redox activity of a hollow internal space [4,118]. Recently, the use of fullerenes has further expanded into more biosensing fields via a combination with nanomaterials such as nitrogen-doped carbon nanotubes (NCNTs) [119], AuNPs [114,120,121,122], and Pt–Pd nanoparticles [123]. These combinations have been shown to be advantageous either because combining the unique properties of these two kinds of nanomaterials or the emergence of new properties leads to novel applications [124].

### 5.1. Synthesis of Fullerenes

Fullerenes can be produced by a laser vaporization technique, in which they are obtained in a supersonic expansion nozzle by applying a pulsed laser onto a graphite target in an inert atmosphere (e.g., helium). This pulsed laser process involves vaporization of carbon from a rotating graphite disc into a high-density helium flow [125]. However, this technique is limited to the production of very small quantities of fullerenes. To overcome this drawback, processes using arc heating of graphite and laser irradiation of poly aromatic hydrocarbon have been developed, which result in the synthesis of large quantities of fullerenes.

In 1990, Krätschmer and Huffman conceptualized the electric arc heating process between graphite rods in an inert atmosphere, in which a fluffy condensate (soot) was generated (Figure 12A) [126]. Fullerenes were then produced by the resistive arc heating technique under atmospheric pressure in a carbon-helium stream, where they were collected on glass shields surrounding the carbon rods. A small amount of toluene was used to extract the fluffy condensate. After extraction, the toluene was removed using a rotary evaporator and the solid mixture, made up of mostly C_60_ with a small amount of higher fullerenes with more than 70 carbon atoms, was purified by liquid chromatography to obtain pure C_60_ [127]. Laser irradiation of polycyclic hydrocarbons (PAHs) has been developed to obtain homologues of fullerenes that may not be obtained in sufficient quantities by the uncontrolled graphite evaporation process. This approach to fullerene synthesis involves the use of PAHs that are composed of multiple aromatic rings. Fullerenes are formed by rolling such unrolled PAH molecules under flash vacuum pyrolysis conditions; a polycyclic aromatic hydrocarbon consisting of 60 carbon atoms transforms into fullerene C_60_ via free radical mechanism when it is irradiated by a laser with a 337 nm wavelength (Figure 12B) [128].

### 5.2. Fullerenes in Enzymatic Biosensing

The effectiveness of enzymatic biosensors is determined by the achievement of an efficient electron transport between redox proteins and electrodes. Due to their complex structures and the deeply buried ligand binding sites of proteins, it is a challenge to achieve direct electron transfer between the redox proteins and the electrode surface [129]. In addition, redox proteins become irreversibly denatured when adsorbed on the electrode surface [119]. In this regard, fullerenes are very beneficial for protein immobilization because they offer a biocompatible microenvironment for this process [130]. Additionally, fullerenes maintain the protein molecule mobility for correctly orienting the redox centers, achieving a proper electron transfer. Furthermore, fullerenes can interact with enzymes via several types of immobilization strategies (e.g., entrapment, encapsulation, covalent binding, cross-linking, and adsorption) that involve covalent or non-covalent bonds [130].

**Figure 13:**
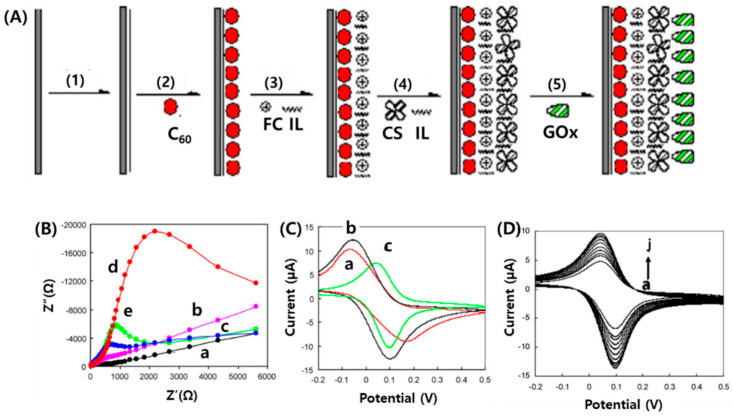
(**A**) General procedure for fabrication of GOx/C_60_-Fc-CS-IL-GCEW. Procedure of the electrode preparation includes five assembly processes, i.e., pretreatment of GCE, immobilization of C_60_, Fc, CS-IL, and GOx on the electrode surface. (**B**) Faradaic impedance spectra that corresponded to (a) bare GCE, (b) C_60_-GCE, (c) C_60_-Fc-GCE, (d) C_60_-Fc-CS-IL-GCE, and (e) GOx/C_60_-Fc-CS-IL-GCE, respectively, in pH 7.0 PBS containing 1.0 mM of [Fe(CN)_6_]^3−/4−^. (**C**) Cyclic voltammograms of (a) Fc-GCE, (b) C_60_-Fc-GCE, and (c) GOx/C_60_-Fc-CS-IL-GCE in pH 7.0 PBS at 100 mV/s. (**D**) Cyclic voltammograms of GOx/C_60_-Fc-CS-IL-GCE in pH 7.0 PBS at 10, 25, 50, 75,100, 200, 300, 400, 500, and 600 mV/s (from a to j), respectively. Reproduced with permission from [116], Copyright @ 2009 Elsevier B.V.

Zhilei et al. [116] prepared a glucose biosensor via the five assembly steps with C_60_, ferrocene (Fc), chitosan (CS) ionic liquid (IL), and glucose oxidase (GOx), respectively, onto a glassy carbon electrode (GCE) surface (Figure 13A). Faradaic impedance for the bare GCE, C_60_-GCE, C_60_-Fc-GCE, C_60_-Fc-IL-CS-GCE, and GOx/C_60_-FcCS-IL-GCE was investigated by electrochemical impedance spectroscopy, the Nyquist plots for which are shown in Figure 13B. The electron transfer resistances of the redox for all modified electrodes are obviously higher than that of the bare electrode, which is because materials modified on the surface of the electrode will partly block the electron transfer of [Fe(CN)_6_]^3−/4−^ solution to the electrode. However, the electron transfer resistance of GOx/C_60_-Fc-CSIL-GCE is remarkably lower than that of C_60_-Fc-IL-CS-GCE. CVs of Fc-GCE, C_60_-Fc-GCE, and GOx/C_60_- Fc-CS-IL-GCE were recorded in pH 7.0 PBS at a scan rate of 100 mV/s (Figure 13C). CVs for modified electrodes showed a pair of redox peaks and synergistic electrocatalytic properties of C_60_, Fc, CS, and IL that led to enhancement of the peak current, signifying the improvement in selectivity and sensitivity of the sensor (Figure 13D). The apparent Michaelis–Menten constant (K_M_) of GOx on the composite medium, 0.03 mM, shows high bio-electrocatalytic activity of the immobilized enzyme toward glucose oxidation. The high performance of this glucose sensor was attributed both to the electrocatalytic activity of C_60_ and Fc and to the network of CS-IL. C_60_ and Fc accelerated the electrochemical reaction and the CS-IL network provided high sensitivity and stability to maintain the enzyme bioactivity and the electron conduction pathways for GOx through Fc and C_60_.

A nanostructured hemoglobin (Hb) biosensor was developed by Lanzellotto et al. [120] by exploiting the beneficial features of a poly fullerene/nitrogen doped-carbon nanotubes (C_60_/NCNTs)/chitosan composite matrix. As an electron mediator, the C_60_ filled within an NCNT could electronically dope the NCNT, thus facilitating the electrochemical reaction of the protein and achieving the fast electron transfer between Hb and the electrode surface. Moreover, this electrochemical platform exhibited excellent electrocatalytic ability toward the reduction of H_2_O_2_ with a linear dynamic range (2.0–225.0 μM). This approach can be easily applied to other redox enzymes or proteins for the determination of H_2_O_2_ in cell extracts or urine.

Barberis et al. [131] demonstrated the simultaneous amperometric detection of ascorbic acid (AA) and antioxidant capacity in fruit juices using fullerenes (C_60_ and C_70_) or SWCNTs and MWCNTs in modified graphite biosensor systems. Their research showed that the combination of fullerene and ascorbate oxidase resulted in complete AA shielding and in the highest selecting capacity toward AA, while nanotubes only increased sensitivity without the ability to discriminate between the AA’s and the phenols’ contribution to the antioxidant capacity. In the AA sensing application, fullerenes played an important role for loading more enzyme and oxidizing more AA on transducer surfaces.

### 5.3. Fullerenes in Affinity Biosensing

**Figure 14:**
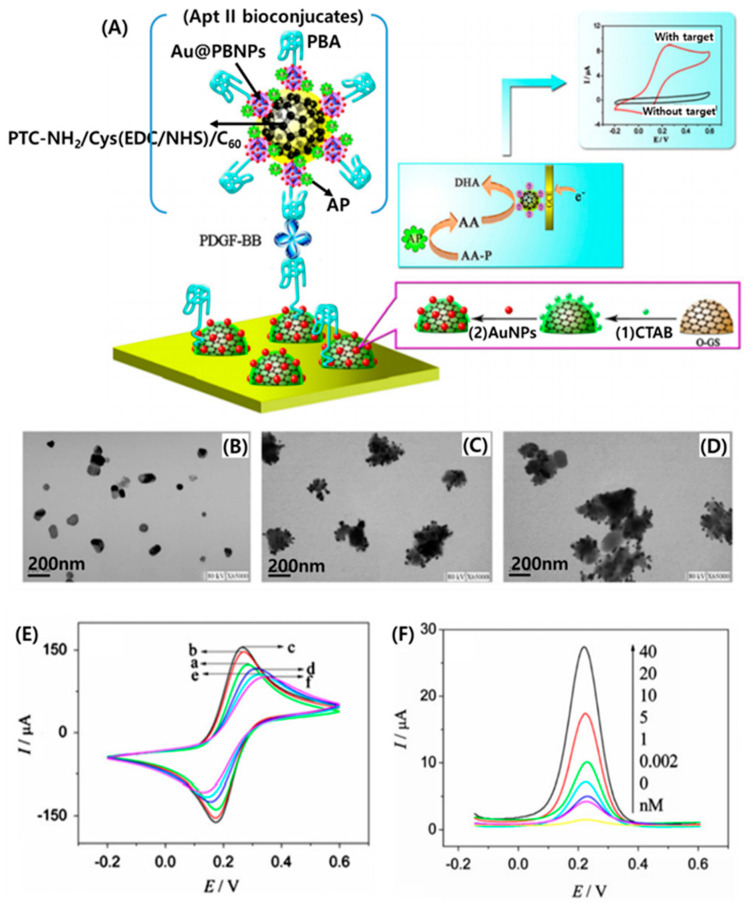
(**A**) Schematic illustration of the stepwise aptasensor fabrication process and the dual signal amplification mechanism. TEM images of (**B**) FC_60_NPs, (**C**) Au@PBNPs, and (**D**) Au@PB/FC_60_. (**E**) Cyclic voltammograms (CVs) of different electrodes in 5.0 mM Fe(CN)_6_^3−/4−^ solution containing 0.1 M KCl at a scan rate of 50 mV/s: (a) bare GCE, (b) O-GS/GCE, (c) AuNPs/O-GS/GCE, (d) Apt I/AuNPs/O-GS/GCE, (e) HT/Apt I/AuNPs/O-GS/GCE, (f) after incubation with 10 nM PDGF. (**F**) DPV responses of the proposed immunosensor after incubation with different concentration of PDGF. Reproduced with permission from [122], Copyright @ 2013 Elsevier B.V.

An electrochemical aptasensing platform for the sensitive and selective detection of small molecules was reported by Han et al. [122]. For the active sensing material, they synthesized C_60_ nanoparticles functionalized by amino and thiol groups (FC_60_NPs) using amino functionalized 3,4,9,10-perylenetetracarboxylic dianhydride (PTC–NH_2_). Prussian Blue carried gold nanoparticles which were decorated on the obtained FC_60_NPs (Au@PB/FC_60_) and were subsequently labeled by detection aptamers and alkaline phosphatase (AP/AptII/Au@PB/FC_60_). It was then possible to use the nanoparticles as tracers in a sandwich aptasensor (Figure 14A).

For the determination of platelet-derived growth factor B-chain (PDGF-BB), an aptasensor was successfully developed using a dual signal amplification strategy by performing biocatalysis of AP toward ascorbic acid 2-phosphate (AA-P) for the in situ production of AA, followed by chemi-catalysis of Au@PB/FC_60_ toward AA to generate dehydroascorbic acid. The sandwich-type aptasensor allowed the determination of PDGF-BB in a linear range between 0.002 and 40 nM with a limit of detection of 0.6 pM. TEM images provide the size and morphology of the as-synthesized samples of FC_60_NPs (Figure 14B), Au@PBNPs (Figure 14C), and Au@PB/FC_60_ (Figure 14D). Electrochemical characteristics of the proposed aptasensor were investigated by cyclic voltammetry. Figure 14E shows CVs of different modified electrodes in 5.0 mM K3Fe(CN)_6_ solution containing 0.1 M KCl at a scan rate of 50 mV/s, including bare GCE, O-GS/GCE, AuNPs/O-GS/GCE, Apt I/AuNPs/O-GS/GCE, HT/Apt I/AuNPs/O-GS/GCE, and after incubation with 10 nM PDGF. The performance of the aptasensor was evaluated by detecting PDGF-BB standard solutions with the addition of 5.0 mM AA-P and the typical DPV obtained for different concentrations of PDGF-BB using AP/Apt II/Au@PB/FC_60_ bioconjugates as tracers (Figure 14F). It is important to note that this aptasensing strategy for highly sensitive and specified target detection opened a new avenue for exploring fullerenes as efficient biomolecule-nanocarriers in the development of electrochemical biosensors.

Furthermore, fullerenes have been shown to also provide a suitable microenvironment for the detection of whole bacterial cells with sensitive and specific identification. Using a C_60_-based biocompatible platform and enzyme functionalized Pt nanochains, Li et al. [114] reported an amperometric sandwich-type immunosensor for the determination of heat-killed *E. coli* O157:H7, which is a pathotype of diarrheagenic *E. coli* and produces one or more Shiga toxins. The immunosensing platform involved a composite of fullerene (C_60_)/ferrocene (Fc)/thiolated chitosan (CHI–SH) and AuNP-coated SiO_2_ nanocomposites (Au–SiO_2_) assembled on the first composite. A large amount of streptavidin (SA) coated on the Au-SiO_2_ surface was used to immobilize biotinylated capture antibodies of *E. coli* O157:H7 (bio-Ab1) through the covalent reaction between biotin and streptavidin. Glucose oxidase-functionalized Pt nanochains (PtNCs) were used to evaluate the signal amplification of antibodies. With a sandwich-type immunoreaction, the detection to the target bacteria ranged from 3.2 × 10^1^ to 3.2 × 10^6^ CFU/mL with a detection limit down to 15 CFU/mL, which is far below the threshold established in clinical diagnosis. These biocompatible platforms, based on the coupling of two different nanostructured materials (fullerene and gold nanoparticles), displayed convenient electrochemical features in terms of good stability and reproducibility, and enhanced electron transfer between the redox center of the protein and the electrode surface.

## 6. Conclusions

CNs have showed tremendous impact on biosensing applications. Owing to their excellent biocompatibility, capability of bioreceptor immobilization, and electrical properties as a transducer, CNs have been used to create efficient, biocompatible biosensors. In addition, functionalization and conjugation with organic compounds or metallic nanoparticles could offer the CNs new properties such as physical, chemical, mechanical, electrical, and optical properties, which would make them even more promising for use in biosensors. As shown in recent studies, mechanical and electrical properties of biosensors have been greatly improved by the combination of two different CNs or their nanocomposites, which could provide for the unique hybridization of the superb properties of individual components or the emergence of new properties in combined nanomaterials. Furthermore, the combination of CNs and biotechnologies has offered a cost-effective solution for rapid target identification based on specific antibody recognition.

Although carbon nanomaterials-based biosensors are promising, they have nonetheless encountered many practical challenges in the field of biological applications. For instance, the fabrication of biosensors usually involves a specific size and helicity of CNs; controlling the size of CNs is difficult during synthesis. In addition, their fabrication is not cost-effective in a large production of nanomaterials with high purity, which is the main reason why the current market prices of CNs are too expensive, thus restricting any realistic commercial applications. In carbon-based biosensors, an enzyme needs to be immobilized onto the surface of carbon nanomaterial-coated electrodes. As a result, the biological receptors may be damaged by their biological activity, biocompatibility, and structural stability. Therefore, developing reversible, reusable, and long-lasting systems to replace current irreversible, disposable, and single-use devices remains a challenge.

## Figures and Tables

**Table 1:** Summary and comparison of three most common CNT synthesis methods.

Method	Arc Discharge	Laser Ablation	CVD
Yield rate	>75%	>75%	>75%
SWCNT or MWCNT	Both	Both	Both
Advantages	Simple, inexpensive, high-quality nanotubes	Relatively high purity, room-temperature synthesis	Simple, low temperature, high purity, large-scale production, aligned growth possible
Disadvantages	High temperature, purification required, tangled nanotubes	Method limited to the lab scale, crude product purification required	Production limited to MWCNTs, defects
